# Targeting Inflammation in Atherosclerosis: Mechanistic Rationale, Clinical Trials, and Regulatory Considerations

**DOI:** 10.31083/RCM52626

**Published:** 2026-08-11

**Authors:** Lazar Mandinov, Chris A. Learn

**Affiliations:** ^1^Cardiovascular Therapeutic Area, Parexel, Newton, MA 02466, USA; ^2^Cell and Gene & Early Phase Therapeutic Area, Parexel, Raleigh, NC 27609, USA

**Keywords:** atherosclerosis, inflammation, interleukin-1β (IL-1β), cardiovascular disease

## Abstract

Atherosclerosis remains the leading cause of global mortality, most commonly manifesting as ischemic heart disease, stroke, and peripheral arterial disease. Although age-adjusted cardiovascular mortality has declined in many developed nations, the global burden of atherosclerotic disease continues to rise due to population aging and growth. This narrative review examines the evolving understanding of atherosclerosis, which has shifted from a predominantly lipid-centric model to a chronic inflammatory disease. Atherosclerosis is initiated by the retention and modification of apolipoprotein B-containing lipoproteins within the arterial wall and propagated by innate and adaptive immune responses, alongside the development of new treatment strategies. Central to this process is activation of the NLRP3 inflammasome and downstream IL-1β–IL-6 signaling and pyroptotic cell death, which amplify vascular inflammation and promote plaque progression and instability. Clinical evidence demonstrates that targeting inflammation, independent of lipid lowering, reduces cardiovascular events, as exemplified by agents such as canakinumab and colchicine. Consequently, the therapeutic landscape is rapidly evolving, with ongoing efforts to refine cytokine-targeted approaches (e.g., IL-6 inhibition), develop selective NLRP3 inhibitors, and advance innovative modalities including cell-based interventions. These strategies aim to provide more durable, precise, and potentially disease-modifying or even curative approaches. However, challenges related to safety, the high cost of biologic agents, and optimal patient selection have limited widespread implementation. A major barrier remains the lack of sensitive and specific biomarkers to identify patients with active vascular inflammation, complicating trial design and therapeutic targeting. In addition to circulating markers such as high-sensitivity C-reactive protein (hsCRP) and IL-6, emerging insights highlight the liver as a central hub linking inflammation and thrombosis through complement and coagulation pathways, offering potential avenues for developing novel biomarkers. Despite promising advances, clinical translation faces persistent challenges, including increased infection risk, inadequate biomarkers for patient selection, cost constraints, and regulatory and payer requirements for hard clinical endpoints. Precision medicine approaches, drug repurposing, and innovative delivery systems may help accelerate development. Ultimately, the management of atherosclerosis is shifting toward a multifaceted approach that integrates lipid-lowering, inflammation resolution, and genetic correction to achieve durable cardiovascular protection.

## 1. Introduction

Atherosclerosis is a chronic arterial disease with three major clinical manifestations—ischemic heart disease, ischemic stroke and peripheral arterial disease. It remains the leading cause of death worldwide [1]. The global crude prevalence of cardiovascular diseases (CVD) is expected to nearly double from 598 million in 2025 to 1.14 billion in 2050, corresponding to a 3.6% year-on-year increase, primarily driven by atherosclerosis [2]. Ischemic heart disease is projected to be the most prevalent CVD in 2050, affecting 471 million people, followed by peripheral artery disease amounting to 236 million cases. Respectively, the global cardiovascular (CV) mortality is projected to increase from 20.5 million deaths in 2025 to 35.6 million deaths in 2050 representing a 73.4% increase (or 2.9% year-on-year) [2].

Notably, the projected increase in the crude prevalence and mortality of CVDs from 2025 to 2050 contrasts with declines in age-standardized CV prevalence (6288 to 6058 per 100,000 population; 3.6% decrease) and age-standardized CV mortality (218 to 151 deaths per 100,000 population; 30.5% decrease), implying that the rise in the total CV mortality will be driven by population growth and the aging population worldwide [2]. The highest age-standardized mortality rates by 2050 are expected in Central Europe, Eastern Europe, and Central Asia, followed by North Africa and the Middle East [2]. Despite progress in reducing age-adjusted mortality of atherosclerotic cardiovascular diseases (ASCVD), the absolute number of people at risk of a CVD-related death continues to rise significantly [3,4]. Together, these findings highlight a critical epidemiologic transition in which demographic forces increasingly offset gains achieved through prevention and treatment. Amid these shifting trends, understanding the early and progressive nature of ASCVD is crucial.

The precise onset of atherosclerotic lesions in humans remains uncertain; however, pathological studies suggest that early signs may emerge as early as infancy [5,6,7,8]. Fatty streaks in the arterial walls gradually evolve into atheromas and characteristic plaques. Acute rupture of these plaques can trigger local thrombosis, resulting in partial or complete occlusion of the affected artery [9]. The clinical consequences depend on the location of the plaque, as well as the extent and speed of vessel occlusion. Atherosclerosis typically has a long latency period and often affects multiple vascular beds simultaneously. Although the “lipid hypothesis” of atherosclerosis dates back more than 120 years [10], it took nearly half a century to establish that lowering plasma cholesterol could prevent the disease—an insight that gained traction with the advent of statins (3-hydroxy-3-methylglutaryl coenzyme A [HMG-CoA] reductase inhibitors) [11]. It is now well understood that hypercholesterolemia contributes to atherosclerosis, and certain plasma lipoproteins, such as low-density lipoproteins (LDL), remnant lipoproteins, and lipoprotein(a) [Lp(a)] are considered atherogenic when present at elevated levels [12].

While the lipid hypothesis laid the foundation for understanding atherosclerosis, recent decades have brought a paradigm shift toward recognizing its chronic inflammatory nature. This paradigm emphasizes the interactions between atherogenic lipoproteins and various vascular wall cells, including endothelial cells, smooth muscle cells, monocytes/macrophages, lymphocytes, and mast cells [13,14,15,16]. One of the earliest events in the arterial intima is the adhesion of circulating monocytes to endothelial cells, followed by their migration into the subintimal space, where they differentiate into macrophages. These macrophages play a critical role in foam cell formation and lesion progression [16].

This narrative review synthesizes the evolving understanding of the inflammatory pathogenesis of atherosclerosis, with a particular emphasis on emerging therapeutic opportunities. Specifically, we focus on the most promising anti-inflammatory strategies currently advancing toward clinical application, including targeted cytokine inhibition, inflammasome-directed therapies, and novel immune-modulatory approaches. This review highlights how emerging anti-inflammatory interventions targeting the inflammasome–Interleukin-1β (IL-1β)–interleukin-6 (IL-6) pro-inflammatory axis are reshaping the treatment paradigm for ASCVD by addressing residual inflammatory risk beyond traditional lipid-lowering. We examine their translational potential, progress in clinical trials, and the regulatory challenges that will influence their path to implementation. Distinct from prior overviews, this work integrates mechanistic insights into inflammation and its resolution with evidence from large-scale clinical studies and evolving regulatory frameworks. Importantly, it provides a clinically grounded perspective on how next-generation therapies may be effectively deployed to mitigate inflammatory risk in practice.

## 2. Residual Inflammatory Risk in Statin‑Treated ASCVD

Despite major advances in lipid-lowering therapy, including high-intensity statins and combination regimens with ezetimibe, proprotein convertase subtilisin/kexin type 9 (PCSK9) inhibitors, or bempedoic acid, a substantial proportion of patients, approximately one-third, remain at elevated risk for recurrent ASCVD events [17,18]. This persistent “residual risk” has catalyzed efforts to identify non-lipid contributors to disease progression. Among these, inflammation has emerged as a central and mechanistically distinct driver of atherosclerosis.

A growing body of clinical evidence demonstrates that targeting inflammatory pathways can significantly reduce cardiovascular event rates, independent of lipid lowering, and may further potentiate the benefits of established therapies [19]. Notably, patients achieving dual control of low-density lipoprotein cholesterol (LDL-C) and inflammatory burden, as measured by high-sensitivity C-reactive protein (hs-CRP), derive the greatest clinical benefit [19]. In this context, hs-CRP has consistently outperformed cholesterol metrics alone in predicting future cardiovascular risk. In an observational analysis of prospectively collected real-world data in 9446 statin-treated patients with LDL-C <70 mg/dL undergoing percutaneous coronary intervention (PCI) for stable coronary or acute coronary syndrome, patients with residual inflammatory risk, defined as hs-CRP ≥2 mg/L, showed a significantly higher hazard of major adverse cardiovascular events (MACE), (hazard ratio [HR], 1.82 (95% confidence interval [CI]: 1.42–2.33), *p* < 0.001), mainly driven by all-cause mortality (HR, 2.81 [95% CI: 1.97–4.02], *p* < 0.001), as compared to patients with no residual risk [18]. While hyperlipidemia and inflammation contribute comparably to risk in untreated individuals, residual inflammatory risk becomes the dominant determinant of outcomes in statin-treated populations, underscoring a critical therapeutic gap.

Importantly, this paradigm does not diminish the foundational role of LDL-C lowering, where a “lower is better” strategy remains strongly supported, but rather highlights the limitations of a lipid-centric approach. Instead, these findings advocate for a more integrated framework that incorporates both inflammatory and metabolic dimensions of risk [20]. This is particularly relevant given that approximately one-third of patients exhibit elevated inflammatory burden and represent a subgroup with heightened comorbidity and worse outcomes, including increased all-cause mortality and spontaneous myocardial infarction (MI) [18].

Collectively, these observations support inflammation as a key determinant of residual cardiovascular risk across the full spectrum of coronary artery disease, encompassing both acute and chronic coronary syndromes. However, critical questions remain regarding the optimal inflammatory targets and the most effective therapeutic strategies, underscoring the need for continued translational and clinical investigation in this space. These questions converge on a central Nucleotide-binding domain, Leucine-rich Repeat, and Pyrin domain-containing protein 3 (NLRP3) inflammasome–IL-1β–IL-6 axis that mechanistically links atherogenic lipoproteins to vascular inflammation, and thus, represents a key focus for emerging therapies.

## 3. NLRP3 Inflammasome–IL-1β–IL-6 Axis in Atherosclerosis: Mechanistic Basis for Targeted Therapies

### 3.1 Modified Lipoproteins as Triggers of Inflammation

A key event in the pathogenesis of atherosclerosis is the transendothelial transport of apolipoprotein B (apoB)-containing lipoproteins. Each atherogenic particle, including LDL, carries a single apoB-100 molecule, which serves as a structural and functional anchor. Although various apoB-containing lipoproteins contribute to plaque formation and progression, LDL has received the most attention due to its abundance among all atherogenic lipoproteins and its role as the primary cholesterol carrier in circulation [21,22,23,24].

**Fig. 1.**
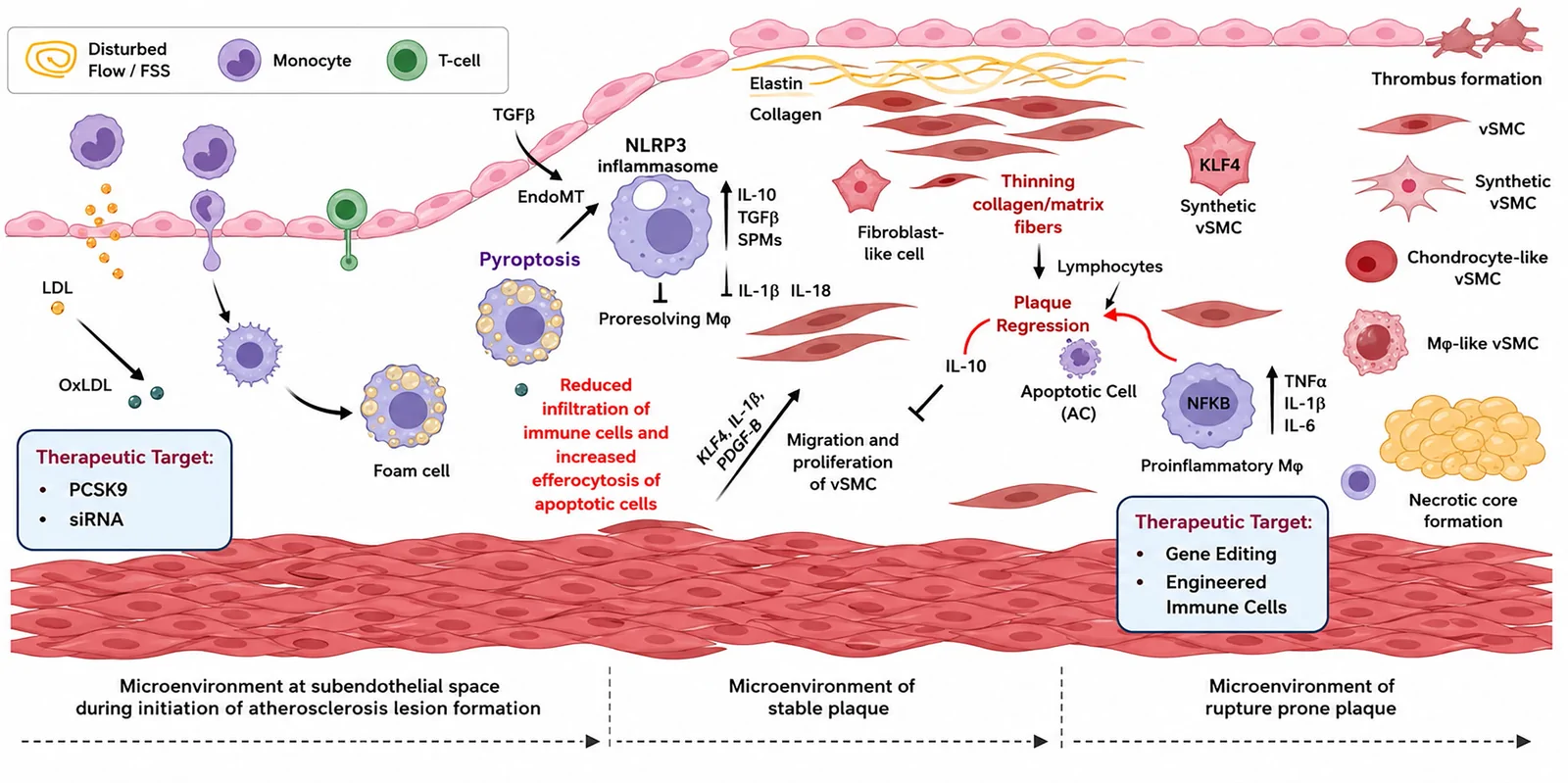
**Atherosclerosis initiation and inflammatory cascade and resolution**. Modified from the original figure published by R. Pandit and A. Yurdagul in Current Atherosclerosis Reports [26] (open access, licensed under a Creative Commons Attribution 4.0 International License). FSS, fluid shear stress; LDL, low-density lipoproteins; OxLDL, oxidized low-density lipoprotein; PCSK9, proprotein convertase subtilisin/kexin type 9; EndoMT, Endothelial-to-mesenchymal transition; NLRP3, Nucleotide-binding domain, Leucine-rich Repeat, and Pyrin domain-containing protein 3; IL-1β, interleukin-1β; SPMs, specialized pro-resolving mediators; KLF4, Kruppel-like factor 4; PDGF-B, platelet-derived growth factor B; vSMC, vascular smooth muscle cell.

According to the “infiltration theory”, atherosclerosis is initiated by the entry and subendothelial retention of LDL and apoB-containing remnants within the arterial wall. ApoB-100 binds to extracellular matrix proteoglycans via ionic interactions [25], trapping LDL particles in the intima—the inner layer of the arterial wall (Fig. 1, Ref. [26]). Once retained, LDL particles are susceptible to a variety of modifications, such as proteolysis, lipolysis, and oxidation [27]. Oxidized LDL particles can aggregate and fuse, and these complexes of lipoprotein particles have a higher affinity for arterial proteoglycans than monomeric lipoproteins, which promotes their retention within the extracellular matrix [28]. These oxidized LDL particles trigger the adaptive immune response by engaging natural immunoglobulin M (IgM) and scavenger receptors, and promoting antibody responses [29,30], but also activate the innate immune system through their adjuvant molecular patterns [31,32]. Inflammatory cells respond by secreting pro-inflammatory cytokines and chemokines, which activate both circulating leukocytes and local endothelial cells. This cascade promotes further recruitment of myeloid-derived inflammatory cells, particularly a subset of monocytes with high inflammatory potential [26,33].

Macrophages, dendritic cells, and vascular smooth muscle cells take up the modified and aggregated LDL, leading to the accumulation of cytoplasmic lipid droplets, and gradually turn into foam cells—a histological hallmark of atherogenesis [34,35]. These modified LDL particles also impair endothelial function by activating NF-κB, increasing vascular permeability, reducing nitric oxide production, upregulating adhesion molecules, and enhancing leukocyte infiltration [36,37]. In addition, intimal macrophages secrete lipoprotein-modifying enzymes, which further alter the retained lipoproteins. In turn, these modified lipoproteins stimulate macrophages to produce even more modifying enzymes, creating a self-perpetuating, pro-atherogenic cycle. This dynamic interplay between lipid accumulation and inflammation drives the progressive development of atherosclerotic plaques [27].

### 3.2 NLRP3 Inflammasome Activation in ASCVD

Accumulation of lipoprotein-derived lipids in macrophages or dendritic cells readily induces activation of a cytosolic multiprotein complex termed the inflammasome [38,39], which acts as a sensor for harmful signals such as DAMP from stressed or injured cells (Fig. 1). These complexes consist of three main components that work together to detect danger. They include a sensor protein that recognizes specific threats, an adaptor protein often called apoptosis-associated speck-like protein containing a caspase-recruitment-domain (ASC), and an effector protein, usually pro-caspase-1. Among the various inflammasomes, the best-studied NLRP3 has been shown to be overexpressed in human atherosclerotic lesions [40,41].

The functional regulation of an active NLRP3 inflammasome is a two-step process. The first step, priming, prepares the cell for action. The triggering signals prime transcription of inflammasome components, including precursor forms of inflammatory signaling molecules such as pro-IL-1β and pro-interleukin-18 (pro-IL-18). Indeed, oxidized LDL has been shown to prime the NLRP3 inflammasome in macrophages in mouse models of atherosclerosis by binding to the cluster of differentiation 36 (CD36)–toll-like receptor (TLR)-4–TLR6 signaling complex on the macrophage surface [42,43]. Once the cell is primed, the same and/or additional signals trigger the assembly and activation of the inflammasome complex (Fig. 1).

In atherosclerotic models, oxidized LDL activates the NLRP3 inflammasome by inducing cholesterol crystallization and ensuing lysosomal damage in macrophages [42,43]. Formation of this complex leads to caspase-1 activation. Caspase-1 cleaves the inactive precursors of IL-1β and IL-18 into their active, secreted forms, which are released from the cell to recruit other immune cells and promote inflammation [44]. This enzyme also plays a role in a distinct form of programmed cell death known as pyroptosis, which involves cell swelling and rupture, contributing to the inflammatory response and helping to clear infected cells [44].

### 3.3 Resolution Biology and Specialized Pro‑Resolving Mediator

Inflammation unfolds in two steps: initiation and resolution. When properly timed and regulated, acute inflammation serves as a protective mechanism [45]. Historically, the resolution phase was considered a passive process, merely involving the breakdown of pro-inflammatory mediators and the dissipation of chemotactic signals. However, it is now recognized as a highly orchestrated and active biological program [26,45]. Resolution of inflammation is mediated by a diverse array of factors, including IL-10, annexin A1, specialized pro-resolving mediators (SPMs) like resolvins, lipoxins, maresins, protectins, and regulatory T cells. Extensive evidence supports the atheroprotective role of IL-10 [4], and similar protective effects have been demonstrated for the SPMs, and annexin A1 [21,46,47]. However, this data is derived from animal or *in vitro* studies; evidence in human atherosclerotic lesions remains limited. Resolution mediators can activate regulatory T cells, which in turn exert potent anti-inflammatory effects and suppress the progression of atherosclerosis [48]. Importantly, the SPMs have been shown to inhibit the priming and to promote the deactivation of the NLRP3 inflammasome in mouse macrophages [26,49]. Resolvin D2 (RvD2) potently inhibited the secretion of mature IL-1β by mouse macrophages *in vitro* and, by the same mechanism, also facilitated the resolution of inflammation *in vivo* [49]. These findings are particularly compelling given IL-1β's role in initiating inflammatory cascades. The resolution phase plays a critical role in curbing leukocyte recruitment, promoting efferocytosis (the clearance of apoptotic cells), facilitating tissue repair, and restoring vascular integrity. Conversely, failure to activate resolution pathways is now recognized as a key driver of persistent inflammation, promoting chronic inflammatory conditions, including atherosclerosis [45,50,51]. While inflammasome activation is critical for combating acute infections, its unchecked or prolonged activity can fuel systemic inflammation and contribute to autoimmune and inflammatory diseases. This dysregulation may stem from genetic predispositions or sustained exposure to inflammatory stimuli.

The accumulation of atherogenic plasma lipoproteins initiates atherogenesis; therefore, their uptake and degradation by intimal macrophages serve as a key protective mechanism, potentially slowing lesion progression. Cholesterol removal from plaques occurs via three main pathways. Efflux from the macrophages to high-density lipoprotein (HDL) particles, enabling reverse transport to the bloodstream. However, this process is often impaired due to dysfunctional foam cells or HDL particles. Migration of foam cells out of the lesion, carrying cholesterol back into circulation. Efferocytosis of aged foam cells by cholesterol-efflux competent macrophages, which may subsequently exit the lesion.

### 3.4 Macrophage Phenotypes and Cell Death

Macrophages in atherosclerotic lesions primarily originate from classical CD14^+^/CD16^–^ monocytes recruited during inflammation [52], where they differentiate into macrophages and contribute to plaque development. Activated endothelial cells and macrophages produce cytokines and adhesion molecules, such as vascular cell adhesion molecule 1 (VCAM-1), intercellular adhesion molecule 1 (ICAM-1), and Endothelial-selectin (E-selectin), which promote further monocyte recruitment. Additionally, “resident” macrophages have been present in the arterial wall since fetal or very early postnatal life [53]. Finally, intimal smooth muscle cells can transform into foam cells and adopt macrophage-like characteristics in response to cholesterol accumulation [54,55].

Within the arterial intima, monocytes differentiate into macrophages and polarize into distinct functional phenotypes in response to their microenvironment, influenced by various combinations and ratios of growth factors, chemokines, and cytokines [33,56]. Two colony-stimulating factors, granulocyte-macrophage colony-stimulating factor (GM-CSF) and macrophage colony-stimulating factor (M-CSF), are widely expressed in the vascular wall [57]. These factors regulate both monocyte-to-macrophage differentiation and the number and function of mature macrophages [58]. GM-CSF and interferon-gamma (IFNγ), produced by activated T-lymphocytes, polarize macrophages toward a pro-inflammatory M1-like phenotype, whereas M-CSF promotes an anti-inflammatory M2-like phenotype. M1 macrophages elaborate pro-inflammatory cytokines such as IL-1α, IL-1β, IL-6, IL-12, IL-15, IL-18, and tumor necrosis factor-α (TNF-α), contributing to atherosclerosis progression. In contrast, anti-inflammatory M2 macrophages release anti-inflammatory cytokines such as IL-4, IL-10, IL-13, and transforming growth factor-β (TGF-β), which play critical roles in resolving inflammation and promoting plaque healing [33,59,60]. Certain interleukins (IL-1β, IL-6, and IL-12) also regulate hepatic C-reactive protein (CRP) production, a key inflammatory biomarker of cardiovascular risk [61,62]. Interestingly, excessive accumulation of cholesteryl ester droplets in highly pro-inflammatory macrophages can shift their phenotype toward fat-storing foam cells with reduced pro-inflammatory potential [63]. However, these foam cells can ultimately become strongly pro-inflammatory upon necrosis when releasing their contents into the plaque extracellular space. Cell death within atherosclerotic lesions occurs via multiple mechanisms [64]. Dead macrophages coalesce into a lipid-rich necrotic core which stimulates the migration of vascular smooth cells into the intima, encapsulated by a collagen fibrous cap, leading to the formation of fibroatheroma [65]. Chronic inflammation leads macrophages to degrade and thin the fibrous cap, forming thin-cap fibroatheromas (TCFA) with caps <65 μm [47,66]. These unstable plaques, characterized by a large necrotic core and thin fibrous cap, are prone to rupture and thrombosis [67].

The inflammatory response varies depending on the type of cell death. Pyroptosis and necroptosis are both pro-inflammatory, but pyroptosis uniquely involves inflammasome activation and requires caspase-1 activity [68]. Pyroptotic cell death is highly pro-inflammatory because it leads to the release of intracellular contents to the extracellular space and the release of IL-1β and IL-18. Necroptosis disrupts the plasma membrane, releasing cellular contents and damage-associated molecular patterns (DAMPs), and plays a role in plaque vulnerability [69]. Modified lipoproteins like oxidized low-density lipoprotein (OxLDL) can induce autophagy, apoptosis, pyroptosis (via NLRP3 inflammasome activation), and necroptosis in cultured macrophages [68,69,70,71].

Conversely, SPMs enhance cell survival and suppress NLRP3 inflammasome activation [49]. Thus, the balance of pro- and anti-inflammatory signals likely influences the dominant form of cell death. Efferocytosis—particularly by macrophages—is a critical mechanism for resolving inflammation. Both SPMs and IL-10 promote efferocytosis [72,73], suggesting that regulatory T cells also enhance this process [48,49]. Regulatory T cells secrete IL-13, which stimulates macrophages to produce and respond to IL-10, a key pro-efferocytic cytokine [49]. IL-13 further supports resolution by increasing collagen production, reducing monocyte recruitment, and skewing macrophages toward a pro-resolving phenotype [74]. Engagement of efferocyte receptors also amplifies anti-inflammatory signaling, promoting the production of RvD1, IL-10, and TGF-β [51]. Taken together, these pathways define discrete nodal points—apoB lipoprotein retention, NLRP3 activation, IL-1β and IL-6 signaling, and defective resolution—that are now being exploited pharmacologically to modify cardiovascular risk.

## 4. Anti-Inflammatory Treatments for ASCVD

Current treatments for atherosclerosis primarily focus on modulating traditional risk factors, such as lipid and glucose-lowering drugs, to prevent and delay plaque formation and progression. Since inflammation plays a pivotal role in the development of atherosclerosis and plaque instability, anti-inflammatory interventions represent novel potential therapies for patients with coronary artery disease or other forms of atherosclerosis. However, not all anti-inflammatory agents and pathways impact cardiovascular outcomes in ASCVD, as evidenced by some failed clinical trials [75,76]. So far, therapeutic agents targeting the inflammasome–IL-1β–IL-6 pro-inflammatory axis represent the most promising strategy for reducing cardiovascular events, with some already approved and others demonstrating encouraging results in ongoing clinical development (Table 1).

**Table 1.** **Development landscape of anti-inflammatory treatments for ASCVD targeting NLRP3–IL-1β–IL-6 inflammatory pathway**.

Pathway	Drug/class	Stage	Key comments
IL-1β	Canakinumab	Completed	Proof-of-concept (CANTOS)
IL-1R	Anakinra	Phase II	Acute inflammation (post-MI)
IL-6 ligand	Ziltivekimab	Phase III	Most advanced active program
IL-6R	Tocilizumab	Phase II	Biomarker/imaging effects
NLRP3	Dapansutrile, DFV890, VTX2735	Phase I–II	Major emerging class
Microtubule NLRP3	Colchicine	Approved	Benchmark anti-inflammatory

CANTOS, Canakinumab Anti-inflammatory Thrombosis Outcomes Study; MI, myocardial infarction.

Early proof of concept was established by trials of cytokine inhibition, most notably the Canakinumab Anti-inflammatory Thrombosis Outcomes Study (CANTOS) trial with canakinumab, which demonstrated that selective suppression of IL-1β reduces recurrent cardiovascular events independently of lipid lowering [77]. Building on this paradigm, colchicine has emerged as a clinically accessible agent, with the COLCOT trial and Low-Dose Colchicine for Secondary Prevention of Cardiovascular Disease 2 (LoDoCo2) trial confirming its ability to lower event rates, likely through broad inhibition of inflammasome activation and leukocyte trafficking [78,79]. Colchicine is the first anti-inflammatory drug with an approved cardiovascular indication in the U.S. and other countries.

### 4.1 IL‑1β Blockade and Colchicine in Outcomes Trial

Canakinumab, a human monoclonal anti-human IL-1β antibody, has anti-inflammatory effects and has been approved for clinical use in juvenile rheumatoid arthritis [80]. The ability of canakinumab to block IL-1β and the downstream inflammatory markers IL-6, TNF-α, and CRP, without affecting lipids or platelets, was demonstrated in a phase 2 study, positioning this drug as the best candidate to further test its independent anti-inflammatory effect in patients with atherosclerosis [61]. The subsequent randomized, double-blind, placebo-controlled CANTOS trial enrolled 10,061 patients with a history of MI and a persistent proinflammatory response, defined as an hs-CRP level ≥2 mg/L [77]. Patients were randomized to either 50 mg, 150 mg, 300 mg canakinumab or placebo. The primary efficacy outcome was the composite of nonfatal MI, nonfatal stroke, and cardiovascular death. It clearly demonstrated that the 150 mg dose of IL-1β inhibitor canakinumab significantly reduced hs-CRP levels from baseline without reducing the LDL cholesterol level and lowered the risk of recurrent cardiovascular events by 15% compared to placebo (HR, 0.85 [95% CI, 0.74–0.98], *p* = 0.02) [77]. Despite its efficacy, the high annual cost (~ $ 200,000) and increased risk of infection limit its widespread use. Nevertheless, CANTOS was a truly landmark trial as it clearly validated the inhibition of the NLRP3 to IL-1 to IL-6 axis as a critical therapeutic target in patients with ASCVD and paved the way for more accessible anti-inflammatory therapies. Anakinra, a recombinant IL-1 receptor antagonist, has been evaluated in multiple ST-elevation MI (STEMI) trials [81]. Pooled VCU ART data on 139 patients showed that there was a marked reduction in death or heart failure (HR 0.16; 95% CI 0.04–0.65), with greater benefit in patients with a high inflammatory burden [81]. However, despite its positive effect on heart failure and acute myocardial infarction, anakirna has never been tested for ASCVD prevention.

Colchicine, an inhibitor of microtubule polymerization, when prescribed in a low dose of 0.5 mg/d orally, also demonstrated that inflammation inhibition safely lowers cardiovascular event rates among statin-treated patients with a magnitude of effect similar to, if not greater than, that of PCSK9 inhibitors [82,83]. Colchicine preferentially accumulates in neutrophils and largely affects neutrophil activity. It has been shown to decrease neutrophil adhesion to inflamed endothelium by reducing L-selectin expression [84]. Colchicine leads to the downregulation of TNFα and suppresses NLRP3 inflammasome activation [36,83]. This leads to a decrease in inflammasome-mediated production of IL-1β and IL-18 and an overall reduction in IL-6 production and CRP [83,85]. The first clinical trial to test colchicine was LoDoCo, an open-label trial, which enrolled 532 patients with stable coronary artery disease and demonstrated a significantly lower rate of acute coronary ischemia, ischemic stroke, and out-of-hospital cardiac arrest (5.3% vs 16%; HR: 0.33; 95% CI: 0.18–0.59; *p* < 0.001) [78]. The subsequent multicenter double-blind randomized LoDoCo2 trial enrolled 5522 patients with clinically stable coronary artery disease on statin therapy [86]. It demonstrated that low-dose colchicine compared to placebo reduced the risk of cardiovascular death, MI, ischemic stroke, or ischemia-driven coronary revascularization by 31% (HR: 0.69; 95% CI: 0.57–0.83; *p* < 0.001) [86]. In addition to patients with stable coronary artery disease, low-dose colchicine was tested in 4745 patients within 30 days of acute myocardial infarction in the COLCOT trial, a double-blind, placebo-controlled study and showed a 23% lower incidence of the primary composite cardiovascular endpoint (HR: 0.77; 95% CI: 0.61–0.96; *p* = 0.02) [79]. A recent meta-analysis including 11,594 patients enrolled in LoDoCo, LoDoCo2, COLCOT, and COPS showed that compared with control, 0.5 mg/d colchicine reduced cardiovascular death, MI, stroke, and urgent coronary revascularization by 32% (HR: 0.68; 95% CI: 0.54–0.81; *p* < 0.0001) [87]. The reduction in the composite endpoint of CV events by colchicine was driven by a statistically significant reduction in the incidence of MI (38%), stroke (62%), and urgent coronary revascularization (44%) [87]. The colchicine trials reemphasized the importance of the inflammatory pathways in atherosclerosis and that reducing the residual inflammatory risk is a second adjunctive approach, alongside lipid-lowering therapy to reduce fatal and nonfatal cardiovascular events [83].

### 4.2 Emerging IL‑6–Targeted Strategies in ASCVD

**Fig. 2.**
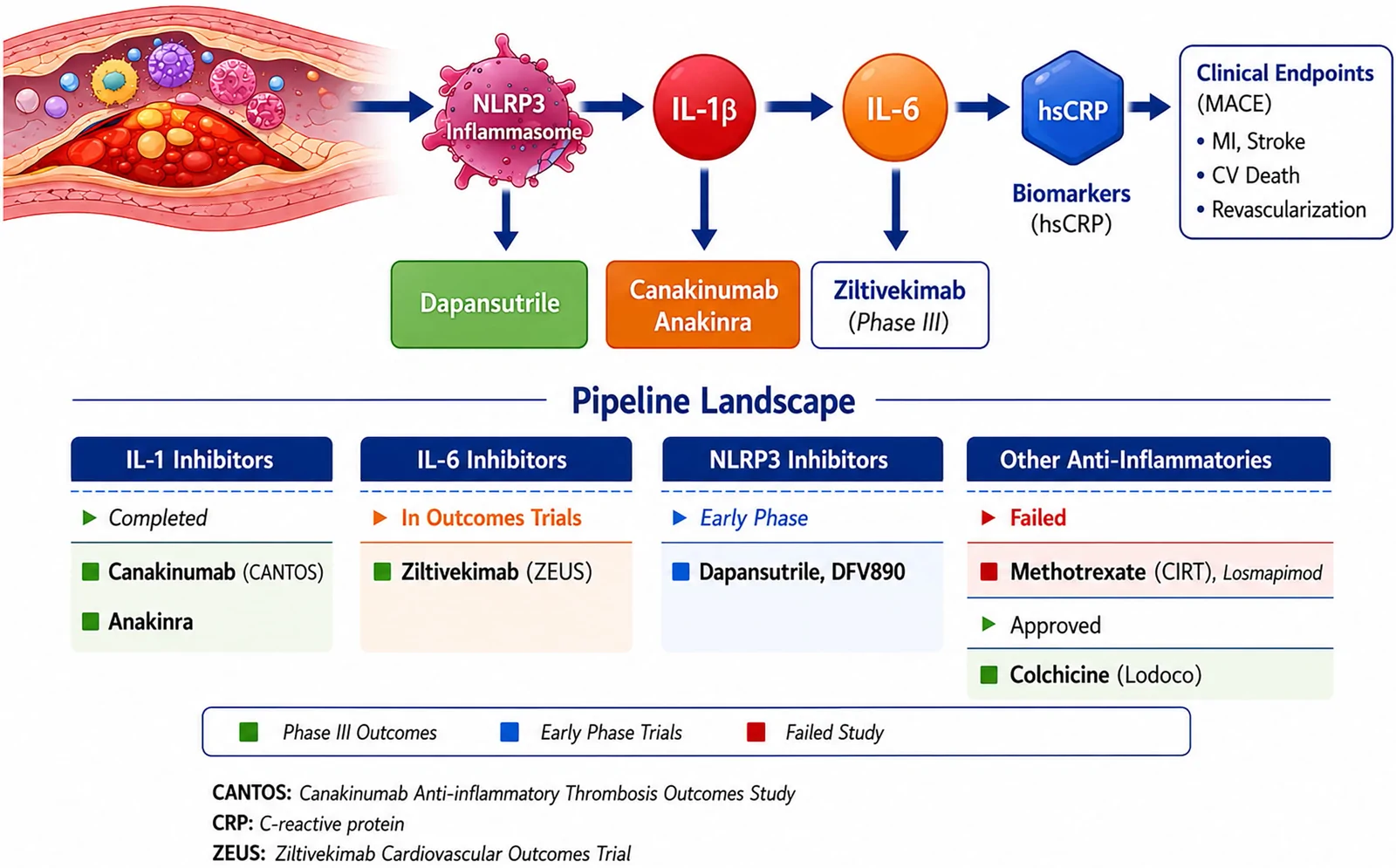
**Inflammasome–IL-1β–IL-6 pro-inflammatory axis and drug pipeline for atherosclerosis**. The figure was created using ChatGTP Classic (Version 26.715.52143). hs-CRP, high-sensitivity C-reactive protein; MACE, major adverse cardiovascular events; CV, cardiovascular; CIRT, Cardiovascular Inflammation Reduction Trial.

The successful clinical trials of anti-inflammatory agents such as canakinumab and colchicine have ushered IL-6 signaling and anti–IL-6 therapeutic strategies to the forefront of modern cardiology. IL-6 inhibition may provide a more selective anti-inflammatory strategy than upstream IL-1β blockade by targeting a central mediator of atherosclerosis-related inflammation. The success of anti-inflammatory treatment in ASCVD has catalyzed a wave of ongoing trials investigating new anti-inflammatory drugs that target key components of the IL-1 to IL-18 cytokine axis, their receptors, and associated kinases and transcription factors [83] (Fig. 2).

Among these agents, ziltivikimab is a monoclonal antibody that directly binds to the IL-6 ligand, effectively blocking both classic signaling (via membrane-bound IL-6 receptor, IL-6R) and trans-signaling (via soluble IL-6R, sIL-6R) without inhibiting IL-6 trans-presentation, a third mode of IL-6 signaling [88]. The ZEUS trial is an ongoing multicenter, double-blind, placebo-controlled, randomized, phase 3 cardiovascular outcomes trial evaluating Ziltivekimab, 15 mg, administered subcutaneously every month or matching placebo in 6376 participants with established ASCVD, CKD, and residual inflammatory risk with hs-CRP ≥2 mg/L [89]. Building on the strong biomarker reductions observed in the phase 2 RESCUE trial—notably profound lowering of hs-CRP—ZUES is designed to test whether selective inhibition of IL-6 signaling translates into a reduction in MACE [89,90]. This trial represents a critical step in validating IL-6 as a central therapeutic node downstream of IL-1β and upstream of hepatic acute-phase responses, with the potential to offer a more targeted and clinically scalable alternative to earlier cytokine-directed approaches.

In contrast to ziltivikimab, antibodies such as tocilizumab, which target the IL-6 receptor itself, are capable of blocking all three IL-6 signaling pathways, classic, trans-signaling, and trans-presentation, offering broader immunomodulatory effects [91,92]. These therapeutic approaches have been used to treat various inflammatory diseases, such as rheumatoid arthritis and systemic lupus erythematosus, as well as other conditions associated with an elevated risk of atherothrombotic events [92]. In a double-blind, placebo-controlled trial of patients with non–ST-elevation myocardial infarction undergoing coronary angiography, a single dose of tocilizumab resulted in a marked attenuation of the inflammatory response and significantly reduced periprocedural troponin release, suggesting a protective effect against myocardial injury, with a favorable safety profile [93]. Extending these findings to STEMI, subsequent studies demonstrated that tocilizumab improved myocardial salvage and reduced microvascular obstruction [94]. However, the reduction in final infarct size did not reach statistical significance, indicating that while IL-6 inhibition favorably influences early injury and tissue-level outcomes, its impact on ultimate infarct burden remains to be fully established.

Despite their promise, the high cost of monoclonal antibody therapies poses a significant barrier to their widespread use in ASCVD prevention, should they prove effective in that context. From a cost-effectiveness standpoint, alternative strategies are being explored. One such candidate is tetrathiomolibdate, a copper chelator, that has demonstrated the ability to inhibit the release of IL-1β, a key pro-inflammatory cytokine implicated in atherogenesis and restenosis [95,96]. If validated in clinical trials, such small-molecule agents could offer a more accessible and scalable approach to inflammation-targeted cardiovascular therapy. Yet even if IL‑1 and IL‑6–directed strategies succeed, a large residual inflammatory burden will likely persist, motivating the development of more upstream interventions such as NLRP3 inhibitors and regenerative cell-based approaches.

## 5. Potential Therapeutic Strategies Addressing Residual Inflammatory Risk in Atherosclerosis

The persistence of residual cardiovascular risk has driven intense research into new and emerging treatment modalities that further address the crucial underlying inflammation and inflammatory cell factors. This new generation of therapies includes innovative strategies aimed at more durable, definitive disease management [97]. While still in clinical development, some of these investigational approaches hold promise to revolutionize treatment by offering the potential for single-dose, long-lasting therapeutic effects.

### 5.1 Oral NLRP3 Inflammasome Inhibitors

Selective inhibition of the NLRP3 inflammasome offers a potentially safer and more comprehensive anti-inflammatory strategy than downstream IL-1 blockade. While agents such as canakinumab have demonstrated clinical efficacy, they are associated with a modest but significant increase in infection risk, likely due to broad suppression of IL-1β signaling downstream of multiple pathogen-sensing inflammasomes [77]. In contrast, targeting the NLRP3 inflammasome preserves redundancy in host defense pathways and may therefore reduce susceptibility to infection. Moreover, NLRP3 inhibition provides broader anti-inflammatory effects by preventing not only IL-1β release but also IL-18 production, pyroptosis, and other pro-inflammatory mediators implicated in sterile inflammation [98]. Consistent with this, preclinical models demonstrate superior efficacy of NLRP3 inhibition compared with IL-1–targeted approaches [99]. These advantages have fueled growing interest in developing selective NLRP3 antagonists; some have failed in early development due to liver toxicity, but several are now advancing through clinical evaluation.

As a selective NLRP3 inflammasome antagonist, dapansutrile is an orally active β-sulfonyl nitrile compound (Fig. 2), that has been identified as a potential treatment for inflammatory conditions [100]. Dapansutrile exerts pleiotropic anti-inflammatory effects that extend beyond direct inhibition of the NLRP3 inflammasome. It also orchestrates the inflammatory milieu by modulating key cytokines such as IL-1β and IL-6, thereby disrupting the chemotaxis and activation of inflammatory cells [101]. These findings support the use of dapansutrile as a promising anti-inflammatory agent and a potential new treatment for ASCVD.

DFV890 (also known as IFM‐2427) is another small molecule designed to inhibit NLRP3 activation and prevent caspase‐1 and subsequent activation of the inflammatory interleukins in response to a broad range of NLRP3‐dependent activators [102]. In a Phase 1 dose-escalation study involving 122 healthy volunteers, DFV890 demonstrated a favorable safety and tolerability profile, along with pharmacokinetic and pharmacodynamic characteristics supportive of target engagement [103]. These findings position DFV890 as a promising oral, potentially first-in-class innate immune modulator that merits further clinical evaluation.

### 5.2 Immunomodulation and Cell‑Based Repair Therapies

In parallel with small-molecule therapies, cell-based therapies offer a distinct yet complementary approach to atherosclerosis management by focusing on tissue repair and regeneration [104,105]. The central premise is that by using the body’s own cells, it is possible to repair damage and restore functionality. Endothelial progenitor cells (EPCs) are a key focus of this research. These cells, mobilized from the bone marrow, help maintain vascular homeostasis and repair damaged vessel linings [106]. Mounting evidence suggests that atherosclerosis is associated with both a reduced number and dysfunction of EPCs [106]. The therapeutic rationale is that administering exogenous EPCs can enhance the body’s natural repair mechanisms, leading to improved endothelial function, stabilization of atherosclerotic plaques, and potentially even disease regression [104,105]. By addressing the physical damage to the arterial wall—the consequence of chronic inflammation and hyperlipidemia—cell therapies can provide a regenerative and restorative benefit that complements the upstream, causal-level interventions of genetic therapies.

The potential of cell therapy is not limited to simple tissue repair. Researchers are also exploring more sophisticated strategies, such as genetically engineering immune cells to create enhanced anti-inflammatory properties [104,105]. The goal is to program these cells to specifically target and resolve inflammation within the atherosclerotic plaque without causing the broad immune suppression that can be a side effect of systemic anti-inflammatory drugs. This targeted approach leverages the natural regulatory and regenerative power of the body’s own cells for a durable therapeutic benefit [104,105]. Cell therapies are designed to reverse the disease or mitigate the consequences of chronic inflammation on the damaged endothelial lining or the atherosclerotic plaque itself. The future of ASCVD treatment may involve a combinatorial approach, where molecular therapy provides long-term, causal correction, while cell therapy offers targeted regenerative and immunomodulatory support for a more complete and lasting therapeutic outcome. This complementary paradigm represents a truly holistic approach to disease management. Realizing the promise of these upstream and regenerative strategies will depend on navigating substantial clinical, regulatory, and economic challenges in a common, chronic disease such as ASCVD.

## 6. Clinical and Regulatory Challenges in Developing Anti‑Inflammatory ASCVD Therapies

Despite growing enthusiasm around anti-inflammatory therapies for atherosclerosis, their clinical development faces a series of formidable challenges. ASCVD is a widespread, chronic condition, and any new therapeutic intervention must meet an exceptionally high safety threshold. This requirement shifts the risk–benefit equation toward demanding substantial, indisputable clinical benefit before regulatory approval or widespread adoption can be considered.

### 6.1 Safety

Safety remains one of the principal barriers to the widespread implementation of inflammation-targeted therapies in ASCVD. Unlike oncology or acute inflammatory diseases, cardiovascular prevention involves chronic treatment of exceptionally large populations over prolonged periods, often decades. Consequently, even relatively infrequent adverse events may translate into substantial absolute morbidity at the population level. The safety threshold for preventive cardiovascular therapeutics is therefore extraordinarily high and fundamentally shapes both regulatory expectations and clinical adoption.

One of the most pressing concerns is the immunosuppressive nature of many anti-inflammatory agents. These drugs, while targeting vascular inflammation, can inadvertently compromise host defenses, increasing the risk of infections, malignancies, and impaired wound healing. These adverse effects are not merely theoretical, as increased infection rates have been documented in multiple randomized controlled trials, including those involving IL-1β inhibitors [77,79]. These findings highlighted a central challenge of chronic immune modulation in ASCVD: inflammatory pathways implicated in atherosclerosis are also essential components of antimicrobial immunity and tissue repair. As a result, long-term cytokine suppression may carry unintended systemic immunologic consequences that become particularly relevant in older cardiovascular populations with multiple comorbidities.

Safety considerations may differ substantially depending on the level of pathway intervention. Downstream cytokine blockade, such as inhibition of IL-1β or IL-6, offers relatively selective targeting but may still interfere with physiologic immune surveillance and acute-phase responses. IL-6 inhibition, for example, may alter hepatic inflammatory signaling, impair infection recognition, and complicate the interpretation of inflammatory biomarkers routinely used in clinical practice. Moreover, hepatotoxicity has been reported in some clinical studies, ultimately leading to discontinuation of further development [107]. Although the occurrence of drug-induced liver injury (DILI) at higher doses and within a short time frame may suggest a component of direct hepatotoxicity, immune-mediated or other mechanisms underlying idiosyncratic DILI cannot be excluded and have to be considered for future drug developments.

The emergence of oral small-molecule NLRP3 inhibitors has generated considerable interest, partly because upstream inflammasome modulation may theoretically provide a more favorable balance between efficacy and systemic immunosuppression. Selective inhibition of pathologic inflammasome activation could potentially attenuate vascular inflammation while preserving broader immune redundancy and host-defense pathways. However, long-term human safety data for these agents remain limited, and important questions persist regarding hepatotoxicity, off-target effects, chronic immune adaptation, and compensatory inflammatory signaling.

### 6.2 Biomarkers

A critical hurdle in the development of anti-inflammatory therapies for ASCVD is the inconsistent detection of residual inflammation and the absence of universally reliable biomarkers. A substantial body of epidemiologic and meta-analytic evidence demonstrates that elevated hs-CRP levels predict myocardial infarction, ischemic stroke, and cardiovascular mortality—even in individuals with well-controlled LDL cholesterol [108]. These findings underscore the concept of “residual inflammatory risk” and highlight the dissociation between lipid burden and inflammatory activity in driving disease progression.

However, neither hs-CRP nor IL-6 reliably identifies all patients with active vascular inflammation. It remains unclear whether this reflects true biological heterogeneity or the limited sensitivity of circulating biomarkers to capture localized vessel wall inflammation. This uncertainty complicates patient selection, making it difficult to define inclusion criteria for clinical trials and to identify those most likely to benefit from anti-inflammatory interventions.

Beyond acute-phase proteins, the liver plays a central role at the intersection of inflammation and thrombosis. It is the primary source of circulating complement components and most coagulation factors. During inflammatory activation, complement cascades generate potent mediators such as C3a and C5a, which exacerbate endothelial dysfunction and promote platelet activation [109]. Concurrently, inflammatory signaling increases hepatic production of procoagulant factors, including fibrinogen and factor VIII, shifting the hemostatic balance toward a prothrombotic state. This convergence of inflammatory and coagulation pathways establishes a self-amplifying cycle of immunothrombosis, increasing the risk of plaque destabilization and atherothrombotic events. Together, these processes position the liver as a critical hub linking systemic inflammation to vascular pathology and as a promising yet underexplored source of novel biomarkers.

### 6.3 Acceptable Endpoints for Regulators and Payers

Regulatory guidance from the U.S. Food and Drug Administration indicates that, despite strong mechanistic evidence linking inflammation to ASCVD, inflammatory biomarkers alone are not currently accepted as surrogate endpoints for drug approval. Biomarkers such as hs-CRP, IL-6, and IL-1β are considered valuable for pharmacodynamic assessment, patient stratification, and trial enrichment, but lack sufficient validation as predictors of clinical benefit. Consequently, regulatory approval for anti-inflammatory therapies in ASCVD continues to rely on demonstration of reduction in hard clinical outcomes, most commonly composite MACE, including myocardial infarction, stroke, coronary revascularization, and cardiovascular death. These endpoints are essential not only for assessing the true risk–benefit profile of novel therapies but also for securing market access and reimbursement. Payers, understandably, will be reluctant to cover expensive new drugs without clear evidence of superiority over existing standards of care. The approval of colchicine for secondary prevention of ASCVD underscores this paradigm, as its efficacy was established through event-driven outcome trials rather than through biomarker modulation. Collectively, these considerations highlight a key translational gap: while inflammation is a validated biological driver of atherosclerosis, its quantitative biomarkers have not yet achieved regulatory acceptance as surrogate endpoints, necessitating large, long-duration outcomes trials for clinical development (Table 2). The development of more refined diagnostic tools, capable of detecting clinically significant vascular inflammation with high fidelity, is urgently needed.

**Table 2.** **Comparison table: biomarkers vs clinical endpoints in ASCVD trials**.

Category	Examples	Regulatory role (FDA)	Utility in trials	Limitations for approval
Inflammatory biomarkers	hsCRP, IL-6, IL-1β, TNF-α	Not accepted as surrogate endpoints	Mechanistic validation; pharmacodynamic readouts; patient enrichment (e.g., residual inflammatory risk)	Lack of validated causal linkage to clinical event reduction; variability; not “reasonably likely” surrogates
Lipid biomarkers	LDL-C, apoB	Accepted surrogate endpoints in specific contexts	Primary endpoints in lipid-lowering trials; regulatory pathway for accelerated approval	Context-dependent; not generalizable to inflammation
Imaging biomarkers	Coronary plaque volume (IVUS), PET vascular inflammation	Supportive/exploratory	Mechanistic insight; early-phase efficacy signals	Not validated as surrogates for ASCVD outcomes
Composite clinical endpoints (MACE)	CV death, MI, stroke, urgent revascularization	Gold standard for approval	Primary endpoints in Phase III trials; event-driven designs	Require large sample size and long follow-up
Individual clinical endpoints	MI, stroke, CV death	Acceptable (often within composites)	Key secondary endpoints; sometimes primary in specific populations	Lower statistical power if used alone
All-cause mortality	Death from any cause	Supportive/confirmatory	High clinical relevance; definitive outcome	Requires very large trials; not always feasible as primary endpoint

hsCRP, high-sensitivity C-reactive protein; apolipoprotein B; apoB, apolipoprotein B; IVUS, intravascular ultrasound; PET, positron emission tomography; FDA, Food and Drug Administration; ASCVD, atherosclerotic cardiovascular diseases.

Compounding these issues is the requirement for anti-inflammatory drugs to demonstrate added value beyond established lipid-lowering therapies such as statins and PCSK9 inhibitors. This raises the bar for efficacy and complicates trial design, as endpoints must reflect incremental benefits in reducing MACE.

### 6.4 Future Translational Strategies

Therapies capable of simultaneously modulating both lipid metabolism and vascular inflammation may offer a particularly attractive translational strategy and should be prioritized in early-phase development. Given the multifactorial nature of ASCVD, future therapeutic success will likely depend on integrated approaches that combine lipid lowering, inflammation control, and improved patient stratification.

Several complementary strategies may help accelerate clinical implementation of inflammation-targeted therapies. Precision medicine: Incorporating genetic profiling, inflammatory biomarkers, and potentially imaging-based assessment of vascular inflammation may improve patient selection and identify subgroups with heightened residual inflammatory risk most likely to benefit from targeted intervention. Such an approach could enhance trial efficiency while minimizing unnecessary exposure in low-risk populations. Drug repurposing represents another promising avenue for accelerating development timelines. Agents with established safety profiles, including colchicine, IL-6 inhibitors such as ziltivekimab, and potentially tetrathiomolybdate, may offer a more rapid path to clinical implementation than entirely novel compounds. A chrono-pharmacology approach that aligns drug administration with circadian inflammatory rhythms may enhance efficacy while reducing systemic toxicity and cumulative immunosuppression.

Localized delivery: Development of tissue-specific formulations could reduce systemic exposure, minimize off-target immune effects, and potentially lower the safety threshold required for chronic administration in preventive cardiovascular populations. Advances in targeted drug delivery may further improve the therapeutic index of anti-inflammatory interventions.

Ultimately, while inflammation-targeted therapies hold substantial promise for transforming ASCVD management, their successful translation will require careful integration of biomarker development, precision diagnostics, innovative trial design, and safer, scalable therapeutic platforms. Together, these strategies may help overcome current scientific, regulatory, and implementation barriers and enable broader adoption of inflammation-directed cardiovascular therapies.

## 7. Limitations

This review has several limitations that should be acknowledged. First, the article was intentionally designed as a focused translational narrative review rather than as a systematic review or meta-analysis. Consequently, the manuscript does not employ predefined inclusion/exclusion criteria, formal evidence grading, or quantitative pooled analyses. The literature selection was therefore guided by mechanistic relevance, translational significance, and clinical impact, with particular emphasis on therapeutic strategies targeting the NLRP3–IL-1β–IL-6 inflammatory axis. As with all narrative reviews, this approach carries an inherent risk of selection bias and may not comprehensively capture all emerging inflammatory pathways or competing therapeutic paradigms in ASCVD.

Second, the review intentionally prioritizes discussion of the NLRP3–IL-1β–IL-6 pathway because it currently represents the most clinically validated inflammation-targeted axis in cardiovascular disease. As a result, other biologically important contributors to atherosclerosis, including TNF-α signaling, interferon pathways, adaptive immune responses, and broader metabolic–immune interactions, are discussed less extensively. The manuscript, therefore, should not be interpreted as a comprehensive overview of all inflammatory mechanisms implicated in ASCVD.

Third, although major clinical outcomes trials such as CANTOS, COLCOT, and LoDoCo2 are discussed in detail, the review was not intended to function as a formal comparative effectiveness analysis. Direct quantitative comparisons between therapeutic agents remain challenging because of substantial heterogeneity in trial populations, baseline inflammatory burden, endpoint definitions, concomitant lipid-lowering therapy, and study designs. Accordingly, the article focuses primarily on mechanistic and translational interpretations rather than on formal cross-trial statistical comparisons or health economic modeling.

Finally, many of the emerging therapeutic approaches discussed, including selective NLRP3 inhibitors, regenerative immunomodulatory strategies, and novel biomarker platforms, remain investigational and lack long-term clinical outcomes data. The field continues to evolve rapidly, and several concepts presented in this review may require substantial refinement as additional mechanistic insights, safety data, and regulatory experience emerge.

## 8. Conclusions

In conclusion, the recognition of atherosclerosis as a chronic inflammatory disease has expanded therapeutic strategies beyond traditional lipid lowering. While reduction of apoB-containing lipoproteins remains the cornerstone of ASCVD prevention, growing evidence indicates that residual inflammatory risk is a distinct and clinically important driver of cardiovascular events. In this context, the NLRP3 inflammasome–IL-1β–IL-6 axis has emerged as the most clinically validated inflammatory pathway in atherosclerosis and a major focus of contemporary drug development.

Landmark trials such as CANTOS, COLCOT, and LoDoCo2 demonstrated that targeting inflammation can reduce cardiovascular events independently of lipid lowering, validating inflammation as a therapeutically actionable process in ASCVD. At the same time, these advances have highlighted major translational challenges, including infection risk, immune dysregulation, biomarker limitations, and the need for large event-driven outcome trials in preventive populations.

Ultimately, inflammation-targeted therapy in ASCVD has evolved from a theoretical concept to a clinically validated therapeutic strategy. However, the field remains in an early translational phase, and continued progress will depend on improved biomarker development, precision patient selection, safer, scalable therapeutic platforms, and deeper understanding of vascular immune biology. Together, these advances have the potential to substantially reshape cardiovascular prevention and improve long-term outcomes in patients with atherosclerotic disease.
